# Distinct Functional Patterns of Gene Promoter Hypomethylation and Hypermethylation in Cancer Genomes

**DOI:** 10.1371/journal.pone.0044822

**Published:** 2012-09-07

**Authors:** Xiaopei Shen, Zheng He, Hongdong Li, Chen Yao, Yang Zhang, Lang He, Shan Li, Jian Huang, Zheng Guo

**Affiliations:** 1 Bioinformatics Centre, School of Life Science, University of Electronic Science and Technology of China, Chengdu, China; 2 College of Bioinformatics Science and Technology, Harbin Medical University, Harbin, China; Ohio State University Medical Center, United States of America

## Abstract

**Background:**

Aberrant DNA methylation plays important roles in carcinogenesis. However, the functional significance of genome-wide hypermethylation and hypomethylation of gene promoters in carcinogenesis currently remain unclear.

**Principal Findings:**

Based on genome-wide methylation data for five cancer types, we showed that genes with promoter hypermethylation were highly consistent in function across different cancer types, and so were genes with promoter hypomethylation. Functions related to “developmental processes” and “regulation of biology processes” were significantly enriched with hypermethylated genes but were depleted of hypomethylated genes. In contrast, functions related to “cell killing” and “response to stimulus”, including immune and inflammatory response, were associated with an enrichment of hypomethylated genes and depletion of hypermethylated genes. We also observed that some families of cytokines secreted by immune cells, such as IL10 family cytokines and chemokines, tended to be hypomethylated in various cancer types. These results provide new hints for understanding the distinct functional roles of genome-wide hypermethylation and hypomethylation of gene promoters in carcinogenesis.

**Conclusions:**

Genes with promoter hypermethylation and hypomethylation are highly consistent in function across different cancer types, respectively, but these two groups of genes tend to be enriched in different functions associated with cancer. Especially, we speculate that hypomethylation of gene promoters may play roles in inducing immunity and inflammation disorders in precancerous conditions, which may provide hints for improving epigenetic therapy and immunotherapy of cancer.

## Introduction

DNA hypermethylation and hypomethylation play important roles in the initiation, progression and metastasis of cancer [1], [2]. It is commonly believed that DNA hypermethylation and hypomethylation are independent processes governed by different mechanisms, and they appear to play separate roles in tumor progression [3], [4]. Specifically, DNA hypermethylation in cancer genomes usually occurs in the promoter regions of tumor suppressor genes, which can result in silencing of tumor suppressor genes [5]. In contrast, DNA hypomethylation often targets DNA repeats, which may induce genomic instability and mutation events in cancer genomes [6], [7], [8], [9]. There is evidence that promoter hypomethylation of some genes may be associated with the development of cancer by regulating the activity of genes [10] and that promoter hypomethylation of specific immunity-related genes may promote carcinogenesis [11], [12]. For example, the promoter hypomethylation of cytokine *IL-10* can activate its expression to inhibit the generation of immune response in breast cancer [11], and the promoter hypomethylation of *SPAN-Xb*, an immunogenic antigen, can induce de novo B-cell response in myeloma cells [12]. However, the biological significance of promoter hypomethylation in cancer is still poorly understood [13].

In this work, we explored the distinct roles of genes with promoter hypermethylation and hypomethylation in cancer (hereafter referred to as hypermethylated and hypomethylated genes for simplicity) using the promoter methylation profiles of five cancer types. First, we evaluated the consistency of functions enriched with hypermethylated (or hypomethylated) genes across different cancer types. Then, we identified hypermethylation-specific (or hypomethylation-specific) functions significantly enriched with hypermethylated genes (or hypomethylated genes) and significantly depleted of hypomethylated genes (or hypermethylated genes). Finally, we discuss potential links between hypomethylated genes in cancer and immune and inflammatory response disorders in precancerous conditions.

## Materials and Methods

### Methylation Data

The promoter methylation datasets for five cancer types were extracted from the Gene Expression Omnibus (GEO) and The Cancer Genome Atlas (TCGA) database (http://tcga-data.nci.nih.gov/tcga), as described in Table 1. For each dataset, the data were derived from paired samples of tumor and adjacent normal tissues from the same organ site, and the percentage of tumor cells in each tumor sample of TCGA was higher than 70% [14]. Details about the preparation of tissues can be found in the TCGA document (http://rcb.cancer.gov/rcb-internet/appl/rfp/07013/SOWAttachmentNo3-BCR-3-10.pdf). To avoid a potential batch effect [15], we selected the batch with the largest sample size for each cancer type for analysis. All data were collected with the Illumina HumanMethylation27 platform, which detected the methylation value of 27578 CpG loci located within the proximal promoter regions of transcription start sites of 14495 genes.

**Table 1:** The methylation data analyzed in this study.

Cancer type	Sample size (cancer vs. normal)	Batch number	Data sources
Colon adenocarcinoma	22∶22	–	GEO:Gse17648 [50]
Kidney renal clear cell carcinoma	50∶50	64	TCGA
Stomach adenocarcinoma	47∶47	48	TCGA
Lung adenocarcinoma	24∶24	58	TCGA
Breast invasive carcinoma	20∶20	93	TCGA

We used level_1 data with methylated signal intensity (M) and unmethylated signal intensity (U). The methylation level (beta-value) for each CpG locus was calculated by max (M, 0)/(|U|+|M|+100), and a constant of 100 was added to regularize the beta value when both U and M values were small [16]. Then, a beta value between 0 (unmethylated) and 1 (fully methylated) was assigned to each CpG locus in each sample. For each dataset, the detection *P* value reported by BeadStudio (Illumina) was used as a quality control measure of probe performance. We excluded samples that consisted of >5% probes with detection *P* values >0.05 and probes that consisted of >10% samples with detection *P* values >0.05. A total of 1092 CpG loci within promoters of 605 sex chromosome genes were also excluded from the analysis to eliminate gender-specific bias.

### Cytokine Data

The cytokine data were derived from the Kyoto Encyclopedia of Genes and Genomes (KEGG) pathway database (ko04052: Cytokines) [17] downloaded on March 8, 2011. The data included 230 cytokines from 8 classes: Class I cytokines (hematopoietin family), Class II cytokines (interferon/IL-10 family), PDGF family, TNF family, IL-1 family, IL-17 family, TGF-beta family and chemokines.

### Selection of Differentially Methylated Genes

The non-parametric Mann-Whitney U test was applied to select differentially methylated (DM) CpG loci around the promoter regions of genes [18] by comparing the beta values of each CpG locus between normal and cancer samples. The false discovery rate (FDR) was controlled by the Benjamin and Hochberg procedure [19]. If the promoter of a gene had both hypermethylated and hypomethylated CpG loci, this gene was excluded from subsequent analyses [20]. The genes with at least one DM CpG locus were termed DM genes. By comparing the mean beta values of DM CpG loci between normal and cancer samples, we classified the DM genes into hypermethylated and hypomethylated genes.

### Functional Enrichment and Consistency Analysis

Using the GO function algorithm [21] with an FDR <0.05, we selected GO terms (biological processes) [22] that were significantly enriched with hypermethylated (or hypomethylated) genes for each cancer type, and then treated the local redundancy. For treatment of local redundancy, when both an ancestor and its offspring term(s) were detected to be statistically significant, the GO function extracted only the ancestor term as being relevant if there was evidence that the remaining genes in the ancestor term were still likely to be relevant to the disease after the removal of genes in its significant offspring term(s), [21]; otherwise, only the offspring term was kept.

If there were N significantly hypermethylated terms in dataset 1, among which K_1_ terms were also identified as significantly hypermethylated in dataset 2, the PO (percentage of overlaps) score of the two term lists (from dataset 1 to dataset 2) was calculated as K_1_/N. Then, we proposed a score, denoted as the POE (percentage of overlaps extended) score, to evaluate the consistency of these two lists of significant GO terms. For a hypermethylated GO term extracted from dataset 1, if its raw P value of enrichment with hypermethylated genes for dataset 2 was lower than 0.05, then it was defined to be tentatively significant in dataset 2. If K_2_ of the N hypermethylated terms extracted from dataset 1 were significant or tentatively significant in dataset 2, the POE score of the two term lists (from dataset 1 to dataset 2) was calculated as K_2_/N. Finally, we performed random experiments to demonstrate that the observed POE score was unlikely to be produced by chance. From dataset 2, we randomly extracted genes as “hypermethylated genes”, with the same number of hypermethylated genes extracted from dataset 2, and then performed the functional analysis and calculated the random POE scores. This process was repeated 10,000 times, and the P value of the observed score from dataset 1 to dataset 2 was calculated as the percentage of the random scores exceeding the observed score. The same analysis was performed for the hypomethylated terms.

## Results

### Extensive Hypermethylation and Hypomethylation of Gene Promoters in Cancers

**Figure 1:**
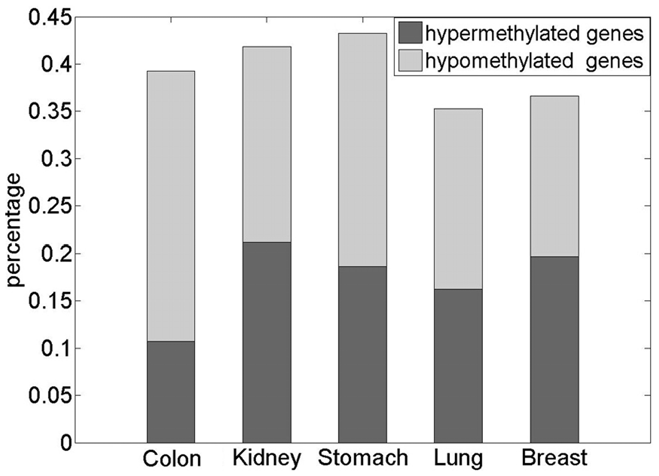
Distributions of DM genes in the five datasets. This figure illustrates the number of DM genes in the datasets for all five cancer types. The x-axis denotes the cancer type, and the y-axis denotes the percentage of DM genes in all detected genes. The light grey and dark grey areas represent hypomethylated and hypermethylated genes, respectively.

We selected DM genes using the Mann-Whitney *U* test with an FDR <5%. As shown in Figure 1, approximately one third of all the measured genes for each dataset were found to be differentially methylated. On average, 56% of the DM genes were hypomethylated in the five cancer types (Figure 1).

### Functional Consistency of Methylation Alterations Across Different Cancer Types

**Figure 2:**
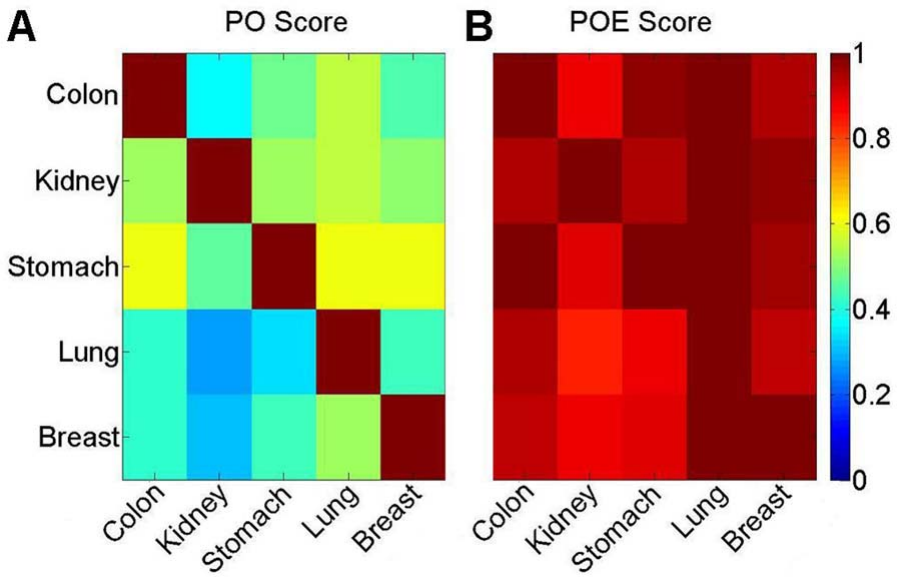
Functional consistency between hypermethylated terms from different cancer types with PO and POE scores. (A) The PO scores of the hypermethylated genes from different cancer types. (B) The POE scores of the hypermethylated genes from different cancer types. Each row represents the scores between the hypermethylated terms for one cancer type and the hypermethylated terms for the other cancer types. A POE score of 1 is shown in red and 0 is indicated in blue.

For each dataset, with an FDR of 5%, the GO-function algorithm [21] was used to identify GO terms that were significantly enriched with hypermethylated genes, called hypermethylated terms. The hypermethylated terms extracted from different datasets appeared to have low PO scores (Figure 2A). For example, only 22 to 24 of the 43 hypermethylated terms extracted from the dataset for kidney carcinoma could be found in the datasets for the other cancer types, with PO scores of 51–55%. However, even for the same cancer type, the significant hypermethylated (or hypomethylated) terms extracted from different datasets tended to have low PO scores due to the inherent limitations of the statistical decision [21]. To address this problem, we proposed the POE score to evaluate the functional consistency of the hypermethylated terms extracted from different datasets (see Materials and Methods). For example, 41 to 43 of the 43 hypermethylated terms extracted for kidney carcinoma had raw enrichment *P* values less than 0.05 in all of the datasets for the other cancer types, with POE scores of 95–100%. However, an average of less than two of the terms extracted for kidney carcinoma had an enrichment *P* value less than 0.05 in 10,000 randomized datasets for each of the other cancer types (see Materials and Methods), which is significantly fewer than the number observed in the original dataset (*P*<0.0001). These results suggested that the re-occurrence in the other four cancer types was not random for the majority of the hypermethylated terms for kidney carcinoma. Similar results were observed for hypermethylated terms extracted for the other four cancer types (Figure 2B). Thus, hypermethylated genes for different cancer types are highly consistent in their function.

With an FDR <0.05, we identified 117 terms that were consistently hypermethylated across the five cancer types. Each of these terms was significant in at least one cancer type and tentatively significant (*P*<0.05) in all of the other four cancer types, which was unlikely to be observed by chance (binomial test, *P*<6.25E–06). As shown in Table S1, these terms are mainly related to “developmental process” (including “cell differentiation” and “cell development”), “transport” (including “calcium ion transport” and “neurotransmitter transport”), “response to stimulus” (including “response to chemical stimulus” and “behavior”) and the “regulation of biological process” (including “regulation of transcription, DNA-dependent” and “regulation of signaling”). Notably, when a term and one of its offspring terms are both detected to be significant, researchers are often interested in the specific offspring term, assuming that specific GO terms might be more biologically relevant [21]. However, in some cases, the general parent term could be globally disturbed. Taking the term “cell differentiation” (GO:0030154) as an example, the genes remaining after the removal of the genes in its four significant offspring terms were still significantly enriched with hypermethylated genes in the dataset for colon adenocarcinoma (hypergeometric test, *P* = 7.98e-005). This result suggested that “regulation of cell differentiation” might be widely disturbed in this cancer.

**Figure 3:**
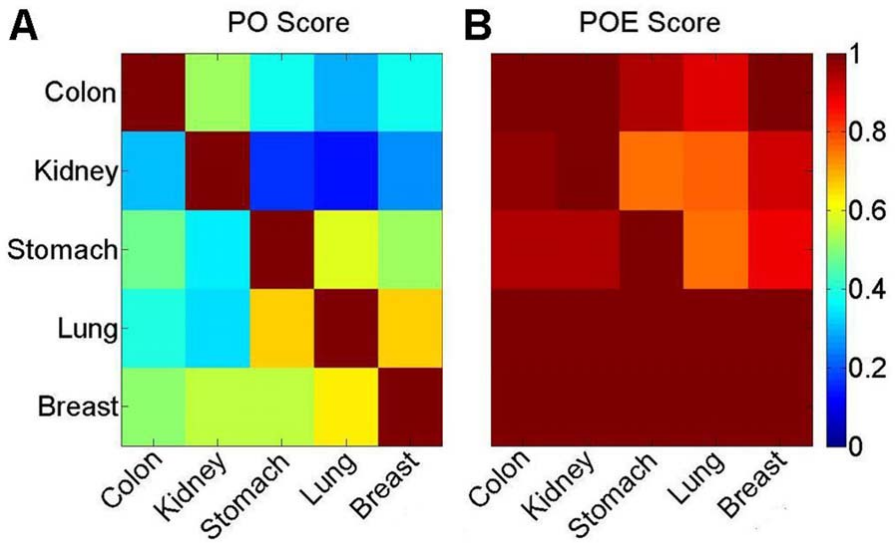
Functional consistency between hypomethylated terms from different cancer types with PO and POE scores. (A) The PO scores of the hypomethylated genes from different cancer types. (B) The POE scores of the hypomethylated genes from different cancer types. Each row represents the scores between the hypomethylated terms for one cancer type and the hypomethylated terms for the other cancer types. A POE score of 1 is shown in red and 0 is indicated in blue.

Similarly, the lists of hypomethylated terms extracted for different cancer types with an FDR of 5% had low percentages of overlap (Figure 3A). For example, only 6 to 11 of the 21 hypomethylated terms extracted from the dataset for colon adenocarcinoma were also found in the datasets for the other cancer types, with PO scores of 28–52%. However, 19 of the 21 hypomethylated terms for colon adenocarcinoma had raw enrichment *P* values less than 0.05 in all of the other four cancer types, and the other two terms had raw enrichment *P* values less than 0.05 in at least one of the other cancer types, all with POE scores greater than 90%. In 10,000 randomized experiments for each cancer type (see Materials and Methods), less than one of the terms, on average, extracted for colon adenocarcinoma had enrichment *P* values less than 0.05 in all of the other four cancer types, which was significantly fewer than the corresponding number observed in the original dataset (*P*<0.0001). Thus, most of the hypomethylated terms for colon adenocarcinoma could be non-randomly found in the datasets for the other four cancer types. Similar results were observed for hypomethylated terms extracted for the other four cancer types (Figure 3B). Therefore, hypomethylated genes for different cancer types were also highly consistent in their function.

Finally, we identified 41 terms that were consistently hypomethylated across the five different cancer types (Table S2). Each of these terms was significant in at least one cancer type and tentatively significant (*P*<0.05) in all of the other cancer types, which was unlikely to be observed by chance (binomial test, *P*<6.25E–06) (see Materials and Methods). These terms were mainly related to “response to stimulus” (including “immune response”, “defense response” and its offspring terms “inflammatory response”, “cellular defense response” and “defense response to bacterium”), and epidermis development (including “keratinocyte differentiation” and its offspring “keratinization”). Taking the term “defense response” as an example, the genes remaining after exclusion of the genes of its three significant offspring terms were still significantly enriched with hypomethylated genes in colon adenocarcinoma (hypergeometric test, *P* = 1.30E–04). Thus, “defense response” might be widely hypomethylated in cancer. As the hypomethylation of genes in “immune response” and “inflammatory response” could be induced by infiltration of lymphocytes in cancer tissue [23], we needed to evaluate the effect of infiltrated lymphocytes on the epigenetic changes of genes annotated in these two terms. Here, we only analyzed the dataset for invasive breast cancer as the data of lymphocyte infiltration in cancer and adjacent normal tissues was available just for this cancer type. We focused on analyzing 7 pairs of tumor and adjacent normal tissues with an equal percentage of lymphocytes in each pair of samples and found that hypomethylated genes were still significantly enriched in “immune response” (*P* = 2.41E–05) but not in “inflammatory response” (*P* = 3.74E–01) which could be due to the low power of detecting hypomethylated genes with an FDR <5% for “inflammatory response” [24]. As the functional enrichment analysis is rather robust to the false discoveries of DM genes [25], we selected hypomethylated genes with an FDR<10% and found that “inflammatory response” was also enriched with hypomethylated genes (*P* = 1.92E–02). These results indicated that the methylation changes in “immune response” as well as in “inflammatory response” could not be explained by the infiltration of lymphocytes in cancer tissue.

Notably, we found that some typical cancer-associated functions such as “cell cycle” and “apoptosis” were not enriched with hypermethylated genes or hypomethylated genes. Oppositely, some of these functions were significantly depleted of both hypermethylated and hypomethylated genes for all five cancer types. For example, “cell cycle” was significantly depleted of both hypermethylated and hypomethylated genes for all five cancer types (all *P*<2.09E–10). This result could be partially due to the strong target gene specificity of methylation alternations [26], [27]. On the other hand, we still observed some genes in these functions that were consistently differentially methylated across the five cancer types. For example, 80 genes associated with cell cycle showed consistent hypermethylation or hypomethylation changes across the five cancer types, indicating that they are also common targets of methylation alternations in these cancer types.

### Hypermethylation- and Hypomethylation-specific Functions

From the 117 terms consistently hypermethylated across the five cancer types, we defined hypermethylation-specific functions as those that were not significantly enriched with hypomethylated genes in any of the cancer types and significantly depleted of hypomethylated genes in at least one cancer type. The depletion analysis was performed using a one-sided hypergeometric distribution test [28]. We found 58 hypermethylation-specific functions, most of which are related to “regulation of biology process” and “developmental process”. Table S3 contains a complete list of hypermethylation-specific functions.

**Figure 4:**
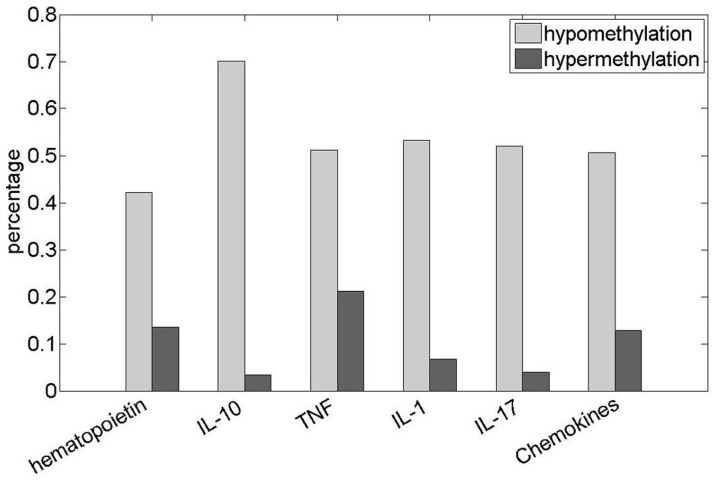
Hypomethylation and hypermethylation of six cytokine gene families. This figure illustrates the percentage of hypomethylated genes and hypermethylated genes in six cytokine gene families. The x-axis denotes the gene family, and the y-axis denotes the percentages of hypomethylated and hypermethylated genes in each of these gene families. The light and dark grey areas represent the hypomethylated and hypermethylated genes, respectively.

Similarly, from the 41 terms consistently hypomethylated across the five cancer types, we defined hypomethylation-specific functions as those that were not significantly enriched with hypermethylated genes in any of the cancer types and significantly depleted of hypermethylated genes in at least one cancer type (Table S4). We found 24 hypomethylation-specific functions, the majority of which are related to response to stimulus (including “immune response”, “response to fungus”, “defense response” and its offspring “inflammatory response”), immune system process and cell killing (Table 2). Considering that immune cells affect malignant cells through the production of various types of cytokines, we found that cell cytokines collected in the KEGG database were significantly hypomethylated in each of the five cancer types (*P* = 3.72E–13, 5.11E–11, 6.60E–08, 2.44E–08 and 5.94e–09 for colon, kidney, stomach, lung and breast cancers, respectively). Specifically, we found that the hematopoietin, *TNF*, *IL1*, *IL10* and *IL17* families of cytokines had a significant tendency to be hypomethylated in all five cancer types. For example, an average of 70.0% of genes in the *IL10* family, which promote innate immune responses from tissue epithelia to limit the damage caused by infection or inflammation [29], were hypomethylated in all five cancer types (Figure 4).

**Table 2:** Hypomethylation-specific functions.

Functional classification	GO-accession #	GO Term
Response to stimulus	GO:0006952	defense response
	GO:0042742	defense response to bacterium
	GO:0006954	inflammatory response
	GO:0051707	response to other organism
	GO:0009617	response to bacterium
	GO:0009620	response to fungus
	GO:0006805	xenobiotic metabolic process
	GO:0006968	cellular defense response
Immune system process	GO:0006955	immune response
	GO:0006959	humoral immune response
	GO:0045321	leukocyte activation
	GO:0002684	positive regulation of immune system process
	GO:0002694	regulation of leukocyte activation
	GO:0046649	lymphocyte activation
Cell killing	GO:0001906	cell killing
	GO:0031640	killing of cells of other organism
Others	GO:0050867	positive regulation of cell activation
	GO:0043903	regulation of symbiosis, encompassing mutualism through parasitism
	GO:0052547	regulation of peptidase activity
	GO:0050994	regulation of lipid catabolic process
	GO:0006909	phagocytosis
	GO:0007606	sensory perception of chemical stimulus
	GO:0007608	sensory perception of smell
	GO:0031424	keratinization

**Figure 5:**
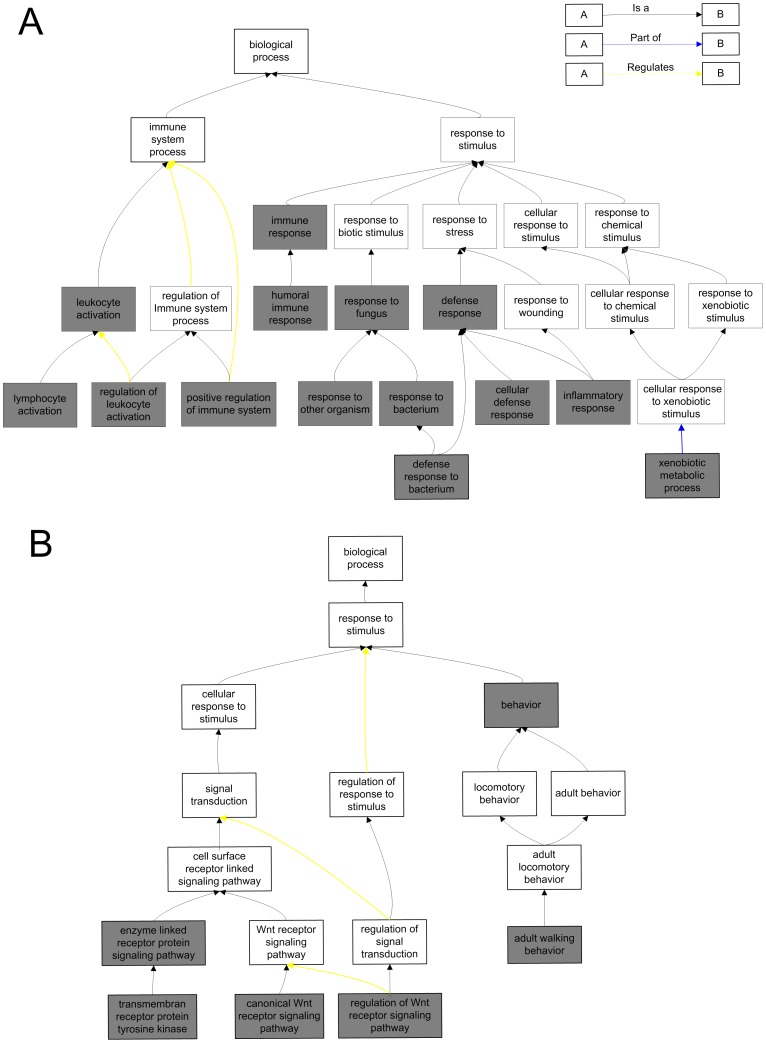
Hypomethylation-specific functions and the hypermethylation-specific functions related to “response to stimulus”. (A) Hypomethylation-specific terms related to “response to stimulus”. (B) Hypermethylation-specific functions related to “response to stimulus”. The significant terms are illustrated with grey.

We noticed that hypermethylation- and hypomethylation-specific functions are related to different types of “response to stimulus”, as shown in Figure 5. The hypomethylation-specific functions are mainly related to “immune response”, “response to fungus” and “defense response” (including its offspring “inflammatory response”), which are mainly performed by immune cells in an organism in response to a potential threat (such as cancer cells and bacteria); in these processes, cells communicate with each other through the use of signal molecules, such as cytokines [30]. In contrast, the hypermethylation-specific functions are mainly related to “signal transduction” within the cell and “behavior” (specific actions or reactions) of an organism in response to external or internal stimuli (Figure 5).

## Discussion

Our results showed that genes with promoter hypermethylation and hypomethylation in different cancer types are highly consistent in function, respectively. Although different tissues have specific methylation patterns [31], this high level of consistency suggests that they have similar methylated functional changes in different cancer types. Our results also indicated that gene promoter hypermethylation and hypomethylation tend to target different biological processes associated with tumor progression. Hypermethylation-specific functions are mostly associated with “development process” and “regulation of biology process”, whereas hypomethylation-specific functions are mostly related to “response to stimulus” (including “immune response”, “response to fungus”, “inflammatory response”), “immune system process” and “cell killing”. These results suggest that DNA hypermethylation and hypomethylation might be independent processes in carcinogenesis [8]. In accordance with previous reports that the methylation state of genes can be modified by environmental stimulus [32], our results showed that both hypermethylated and hypomethylated functions are related to “response to stimulus”. Specifically, our results further revealed that hypermethylation and hypomethylation are associated with different types of “response to stimulus”. Notably, if a function is significantly enriched with hypomethylated (or hypermethylated) genes, it indicates that a significant portion of genes in this function are hypomethylated (or hypermethylated) in cancer, however, it does not mean that this function cannot include a small number of hypermethylated (or hypomethylated) genes. For example, in the hypomethylation-specific function “immune response”, *IRF4*, which negatively regulates toll-like-receptor signalling that is central to the activation of innate and adaptive immune systems [33], was observed to be hypermethylated in all five cancer types. We also found that the “G-protein coupled receptor protein signaling pathway” was significantly enriched with both hypermethylated genes and hypomethylated genes across all five cancer types. These results may be due to the hypomethylation of chemokine receptors and the hypermethylation of genes related to the signals transduction within the cell, both of which may disturb pathways contributing to carcinogenesis [34].

Although our results showed that genes with promoter methylation alternations in different cancer types are highly consistent in their function, cancer is a highly heterogeneous disease with respect to different DM genes in different patients. Even for the same cancer type, unique subtypes are characterized by distinct epigenetic alternations [35], [36], [37], which, should also be consistent in function. For example, we found that the four lists of hypermethylated genes for the four colon cancer subtypes (CIMP-H, CIMP-L, cluster 3 and cluster 4) reported by Hinoue et al [35] were highly consistent in function (Figure S1) although these subtypes were differ in terms of their hypermethylated genes [35]. Specifically, for the 117 terms consistently hypermethylated across different cancer types, we found that 115 terms were consistently enriched with hypermethylated genes for all four subtypes (hypergeometric test, *P*<0.05) and the other two terms were also marginally significant for all four subtypes (hypergeometric test, *P*<0.1). Different samples for a particular cancer type may harbor different methylation alternations which could also be consistent in function.

To extrapolate the functional consequence of methylation alternations of gene promoters in cancer genomes, researchers often investigate the relationship between gene methylation and gene expression. Hypermethylation of gene promoters is significantly correlated with the down-regulation of gene expression but hypomethylation of gene promoters is not or is only weakly correlated with gene up-regulation [38], [39]. Similar complex relationships were also observed at the functional level. To exemplify this, we analyzed 16 of the 20 pairs of samples for invasive breast cancer that contained both methylation and expression data. The differentially expressed genes were selected using the SAM (significance analysis of microarray) algorithm [40] with an FDR <0.05. Then, for the 117 terms consistently hypermethylated across the five cancer types, we found that 64 terms were significantly enriched with down-regulated genes (hypergeometric test with an FDR <0.05) and 83 terms were marginally enriched with down-regulated genes (hypergeometric test with *P*<0.1). However, for the 41 terms that were consistently hypomethylated across the five cancer types, we found that none was enriched with up-regulated genes. Oppositely, 10 hypomethylated terms (including “inflammatory response” and “leukocyte migration”) were even enriched with down-regulated genes. One possible explanation for this phenomenon is the hypothesis that hypomethylation of gene promoters must cooperate with other key activators such as appropriate levels of transcriptional factors [37], [41] to control gene expression. For example, as inflammatory genes tend to be hypomethylated in inflammatory diseases [42], [43], [44], we could hypothesize that hypomethylation of inflammatory gene promoters may happen in precancerous inflammatory disorders, which together with the activation of the coupled activators could induce hyperactivation of inflammatory response in the precancerous conditions. During the development of cancer, the hypomethylation of inflammatory genes could be inherited through cell division, whereas the coupled activators could lose function due to genome instability induced by the pro-tumorigenic microenvironment [45], [46], [47]. Thus we could observe these genes’ down-regulation coexisting with hypomethylation in cancer. To prove this hypothesis, we need to monitor the methylation and expression changes during the progression from precancerous inflammation to cancer, which is a difficult task but deserves future study.

Insight into the functional roles of DNA methylation alterations in cancer genomes may help improve the epigenetic therapy of cancer. Currently, most epigenetic drugs are hypomethylating agents that target hypermethylated genes in cancer [48]. However, because promoter hypomethylation of genes may also play an important role in carcinogenesis, agents targeting hypomethylated genes in cancer might be useful for cancer therapy. For example, reversal of the hypomethylation status of urokinase (*uPA*) promoter blocks breast cancer growth and metastasis [49]. Considering the close link between promoter hypomethylation and immunity, epigenetic therapy and immunotherapy may need to be combined for the treatment of cancer.

## Supporting Information

Figure S1 **The POE scores between every two lists of the hypermethylated functions extracted for the four methylation-based subtypes of colon cancer.** Each row represents the scores between the hypermethylated terms for one subtype and the hypermethylated terms for the other subtypes. The POE score 1 is shown in red and 0 is indicated in blue. The details of the four subtypes are described in [35].
(TIF)

Table S1 ***Terms that are consistently hypermethylated across different cancer types.*** *This table shows the 117 GO terms that are consistently hypermethylated across different cancer types with the P values of enrichment for all of the cancer types.*
(XLS)

Table S2 ***Terms that are consistently hypomethylated across different cancer types.*** *This table shows the 41 GO terms that are consistently hypomethylated across different cancer types with the P values of enrichment for all of the cancer types.*
(XLS)

Table S3 ***Hypermethylation-specific functions.*** *This table shows the 58 hypermethylation-specific functions with the P values of depletion for all of the cancer types.*
(XLS)

Table S4 ***Hypomethylation-specific functions.*** *This table shows the 24 hypomethylation-specific functions with the P values of depletion for all of the cancer types.*
(XLS)

## References

[1] Ting AH, McGarvey KM, Baylin SB (2006) The cancer epigenome–components and functional correlates. Genes Dev 20: 3215–3231. 17158741 10.1101/gad.1464906

[2] Jones PA, Baylin SB (2007) The epigenomics of cancer. Cell 128: 683–692. 17320506 10.1016/j.cell.2007.01.029 PMC3894624

[3] Frigola J, Sole X, Paz MF, Moreno V, Esteller M, et al (2005) Differential DNA hypermethylation and hypomethylation signatures in colorectal cancer. Hum Mol Genet 14: 319–326. 15574462 10.1093/hmg/ddi028

[4] Ehrlich M, Jiang G, Fiala E, Dome JS, Yu MC, et al (2002) Hypomethylation and hypermethylation of DNA in Wilms tumors. Oncogene 21: 6694–6702. 12242669 10.1038/sj.onc.1205890

[5] Herman JG, Baylin SB (2003) Gene silencing in cancer in association with promoter hypermethylation. N Engl J Med 349: 2042–2054. 14627790 10.1056/NEJMra023075

[6] Ferguson AT, Vertino PM, Spitzner JR, Baylin SB, Muller MT, et al (1997) Role of estrogen receptor gene demethylation and DNA methyltransferase.DNA adduct formation in 5-aza-2'deoxycytidine-induced cytotoxicity in human breast cancer cells. J Biol Chem 272: 32260–32266. 9405430 10.1074/jbc.272.51.32260

[7] Ehrlich M (2002) DNA hypomethylation, cancer, the immunodeficiency, centromeric region instability, facial anomalies syndrome and chromosomal rearrangements. J Nutr 132: 2424S–2429S. 12163705 10.1093/jn/132.8.2424S

[8] Ehrlich M, Woods CB, Yu MC, Dubeau L, Yang F, et al (2006) Quantitative analysis of associations between DNA hypermethylation, hypomethylation, and DNMT RNA levels in ovarian tumors. Oncogene 25: 2636–2645. 16532039 10.1038/sj.onc.1209145 PMC1449872

[9] Cash HL, Tao L, Yuan JM, Marsit CJ, Houseman EA, et al.. (2011) LINE-1 hypomethylation is associated with bladder cancer risk among nonsmoking Chinese. Int J Cancer. 10.1002/ijc.26098 PMC3208798 21445976

[10] Pulukuri SM, Estes N, Patel J, Rao JS (2007) Demethylation-linked activation of urokinase plasminogen activator is involved in progression of prostate cancer. Cancer Res 67: 930–939. 17283123 10.1158/0008-5472.CAN-06-2892 PMC1832148

[11] Son KS, Kang HS, Kim SJ, Jung SY, Min SY, et al (2010) Hypomethylation of the interleukin-10 gene in breast cancer tissues. Breast 19: 484–488. 20646924 10.1016/j.breast.2010.05.011

[12] Wang Z, Zhang J, Zhang Y, Lim SH (2006) SPAN-Xb expression in myeloma cells is dependent on promoter hypomethylation and can be upregulated pharmacologically. Int J Cancer 118: 1436–1444. 16187275 10.1002/ijc.21499

[13] Ehrlich M (2009) DNA hypomethylation in cancer cells. Epigenomics 1: 239–259. 20495664 10.2217/epi.09.33 PMC2873040

[14] The Cancer Genome Atlas Research Network (2011) Integrated genomic analyses of ovarian carcinoma. Nature 474: 609–615. 21720365 10.1038/nature10166 PMC3163504

[15] Leek JT, Scharpf RB, Bravo HC, Simcha D, Langmead B, et al (2010) Tackling the widespread and critical impact of batch effects in high-throughput data. Nat Rev Genet 11: 733–739. 20838408 10.1038/nrg2825 PMC3880143

[16] Bibikova M, Lin Z, Zhou L, Chudin E, Garcia EW, et al (2006) High-throughput DNA methylation profiling using universal bead arrays. Genome Res 16: 383–393. 16449502 10.1101/gr.4410706 PMC1415217

[17] Kanehisa M, Goto S (2000) KEGG: kyoto encyclopedia of genes and genomes. Nucleic Acids Res 28: 27–30. 10592173 10.1093/nar/28.1.27 PMC102409

[18] Anisowicz A, Huang H, Braunschweiger KI, Liu Z, Giese H, et al (2008) A high-throughput and sensitive method to measure global DNA methylation: application in lung cancer. BMC Cancer 8: 222. 18673580 10.1186/1471-2407-8-222 PMC2546425

[19] Benjamini Y, Hochberg Y (1995) Controlling the false discovery rate: a practical and powerful approach to multiple testing. Journal of the royal statistical society Series B (Methodological) 57: 289–300.

[20] Kim EH, Park AK, Dong SM, Ahn JH, Park WY (2010) Global analysis of CpG methylation reveals epigenetic control of the radiosensitivity in lung cancer cell lines. Oncogene 29: 4725–4731. 20531302 10.1038/onc.2010.223

[21] Wang J, Zhou X, Zhu J, Gu Y, Zhao W, et al.. (2011) GO-function: deriving biologically relevant functions from statistically significant functions. Brief Bioinform. 10.1093/bib/bbr041 21705405

[22] Ashburner M, Ball CA, Blake JA, Botstein D, Butler H, et al (2000) Gene ontology: tool for the unification of biology. The Gene Ontology Consortium. Nat Genet 25: 25–29. 10802651 10.1038/75556 PMC3037419

[23] Dedeurwaerder S, Desmedt C, Calonne E, Singhal SK, Haibe-Kains B, et al (2011) DNA methylation profiling reveals a predominant immune component in breast cancers. EMBO Mol Med 3: 726–741. 21910250 10.1002/emmm.201100801 PMC3377115

[24] Pawitan Y, Michiels S, Koscielny S, Gusnanto A, Ploner A (2005) False discovery rate, sensitivity and sample size for microarray studies. Bioinformatics 21: 3017–3024. 15840707 10.1093/bioinformatics/bti448

[25] Zou J, Hao C, HE L, Guo Z (2012) Identifying disease-associated functions based on weak signals of differential expressions of genes. ACTA Biophysica sinica 28: 232–241.

[26] Ushijima T, Asada K (2010) Aberrant DNA methylation in contrast with mutations. Cancer Sci 101: 300–305. 19958364 10.1111/j.1349-7006.2009.01434.x PMC11159270

[27] Feltus FA, Lee EK, Costello JF, Plass C, Vertino PM (2003) Predicting aberrant CpG island methylation. Proc Natl Acad Sci U S A 100: 12253–12258. 14519846 10.1073/pnas.2037852100 PMC218745

[28] Rivals I, Personnaz L, Taing L, Potier MC (2007) Enrichment or depletion of a GO category within a class of genes: which test? Bioinformatics 23: 401–407. 17182697 10.1093/bioinformatics/btl633

[29] Ouyang W, Rutz S, Crellin NK, Valdez PA, Hymowitz SG (2011) Regulation and functions of the IL-10 family of cytokines in inflammation and disease. Annu Rev Immunol 29: 71–109. 21166540 10.1146/annurev-immunol-031210-101312

[30] Miyajima A, Kitamura T, Harada N, Yokota T, Arai K (1992) Cytokine receptors and signal transduction. Annu Rev Immunol 10: 295–331. 1590989 10.1146/annurev.iy.10.040192.001455

[31] Illingworth R, Kerr A, Desousa D, Jorgensen H, Ellis P, et al (2008) A novel CpG island set identifies tissue-specific methylation at developmental gene loci. PLoS Biol 6: e22. 18232738 10.1371/journal.pbio.0060022 PMC2214817

[32] Jaenisch R, Bird A (2003) Epigenetic regulation of gene expression: how the genome integrates intrinsic and environmental signals. Nat Genet 33 Suppl: 245–254 10.1038/ng1089 12610534

[33] O’Neill LA (2008) When signaling pathways collide: positive and negative regulation of toll-like receptor signal transduction. Immunity 29: 12–20. 18631453 10.1016/j.immuni.2008.06.004

[34] Dorsam RT, Gutkind JS (2007) G-protein-coupled receptors and cancer. Nat Rev Cancer 7: 79–94. 17251915 10.1038/nrc2069

[35] Hinoue T, Weisenberger DJ, Lange CP, Shen H, Byun HM, et al (2012) Genome-scale analysis of aberrant DNA methylation in colorectal cancer. Genome Res 22: 271–282. 21659424 10.1101/gr.117523.110 PMC3266034

[36] Weisenberger DJ, Siegmund KD, Campan M, Young J, Long TI, et al (2006) CpG island methylator phenotype underlies sporadic microsatellite instability and is tightly associated with BRAF mutation in colorectal cancer. Nat Genet 38: 787–793. 16804544 10.1038/ng1834

[37] Noushmehr H, Weisenberger DJ, Diefes K, Phillips HS, Pujara K, et al (2010) Identification of a CpG island methylator phenotype that defines a distinct subgroup of glioma. Cancer Cell 17: 510–522. 20399149 10.1016/j.ccr.2010.03.017 PMC2872684

[38] Yao C, Li H, Shen X, He Z, He L, et al (2012) Reproducibility and concordance of differential DNA methylation and gene expression in cancer. PLoS One 7: e29686. 22235325 10.1371/journal.pone.0029686 PMC3250460

[39] Sadikovic B, Yoshimoto M, Chilton-MacNeill S, Thorner P, Squire JA, et al (2009) Identification of interactive networks of gene expression associated with osteosarcoma oncogenesis by integrated molecular profiling. Hum Mol Genet 18: 1962–1975. 19286668 10.1093/hmg/ddp117

[40] Tusher VG, Tibshirani R, Chu G (2001) Significance analysis of microarrays applied to the ionizing radiation response. Proc Natl Acad Sci U S A 98: 5116–5121. 11309499 10.1073/pnas.091062498 PMC33173

[41] Hoffmann MJ, Schulz WA (2005) Causes and consequences of DNA hypomethylation in human cancer. Biochem Cell Biol 83: 296–321. 15959557 10.1139/o05-036

[42] Nimmo ER, Prendergast JG, Aldhous MC, Kennedy NA, Henderson P, et al (2012) Genome-wide methylation profiling in Crohn’s disease identifies altered epigenetic regulation of key host defense mechanisms including the Th17 pathway. Inflamm Bowel Dis 18: 889–899. 22021194 10.1002/ibd.21912

[43] Qiu W, Baccarelli A, Carey VJ, Boutaoui N, Bacherman H, et al (2012) Variable DNA methylation is associated with chronic obstructive pulmonary disease and lung function. Am J Respir Crit Care Med 185: 373–381. 22161163 10.1164/rccm.201108-1382OC PMC3297093

[44] Cooke J, Zhang H, Greger L, Silva AL, Massey D, et al.. (2012) Mucosal genome-wide methylation changes in inflammatory bowel disease. Inflamm Bowel Dis. 10.1002/ibd.22942 22419656

[45] Richards KL, Zhang B, Baggerly KA, Colella S, Lang JC, et al (2009) Genome-wide hypomethylation in head and neck cancer is more pronounced in HPV-negative tumors and is associated with genomic instability. PLoS One 4: e4941. 19293934 10.1371/journal.pone.0004941 PMC2654169

[46] Colotta F, Allavena P, Sica A, Garlanda C, Mantovani A (2009) Cancer-related inflammation, the seventh hallmark of cancer: links to genetic instability. Carcinogenesis 30: 1073–1081. 19468060 10.1093/carcin/bgp127

[47] de Visser KE, Eichten A, Coussens LM (2006) Paradoxical roles of the immune system during cancer development. Nat Rev Cancer 6: 24–37. 16397525 10.1038/nrc1782

[48] Yoo CB, Jones PA (2006) Epigenetic therapy of cancer: past, present and future. Nat Rev Drug Discov 5: 37–50. 16485345 10.1038/nrd1930

[49] Pakneshan P, Szyf M, Farias-Eisner R, Rabbani SA (2004) Reversal of the hypomethylation status of urokinase (uPA) promoter blocks breast cancer growth and metastasis. J Biol Chem 279: 31735–31744. 15150277 10.1074/jbc.M401669200

[50] Kim YH, Lee HC, Kim SY, Yeom YI, Ryu KJ, et al (2011) Epigenomic analysis of aberrantly methylated genes in colorectal cancer identifies genes commonly affected by epigenetic alterations. Ann Surg Oncol 18: 2338–2347. 21298349 10.1245/s10434-011-1573-y PMC3393129

