# Supplementary figures and images for: Distinct Functional Patterns of Gene Promoter Hypomethylation and Hypermethylation in Cancer Genomes
Source: PLoS One. 2012 Sep 7;7(9):e44822. doi: 10.1371/journal.pone.0044822 (PMC3436878; doi:10.1371/journal.pone.0044822)

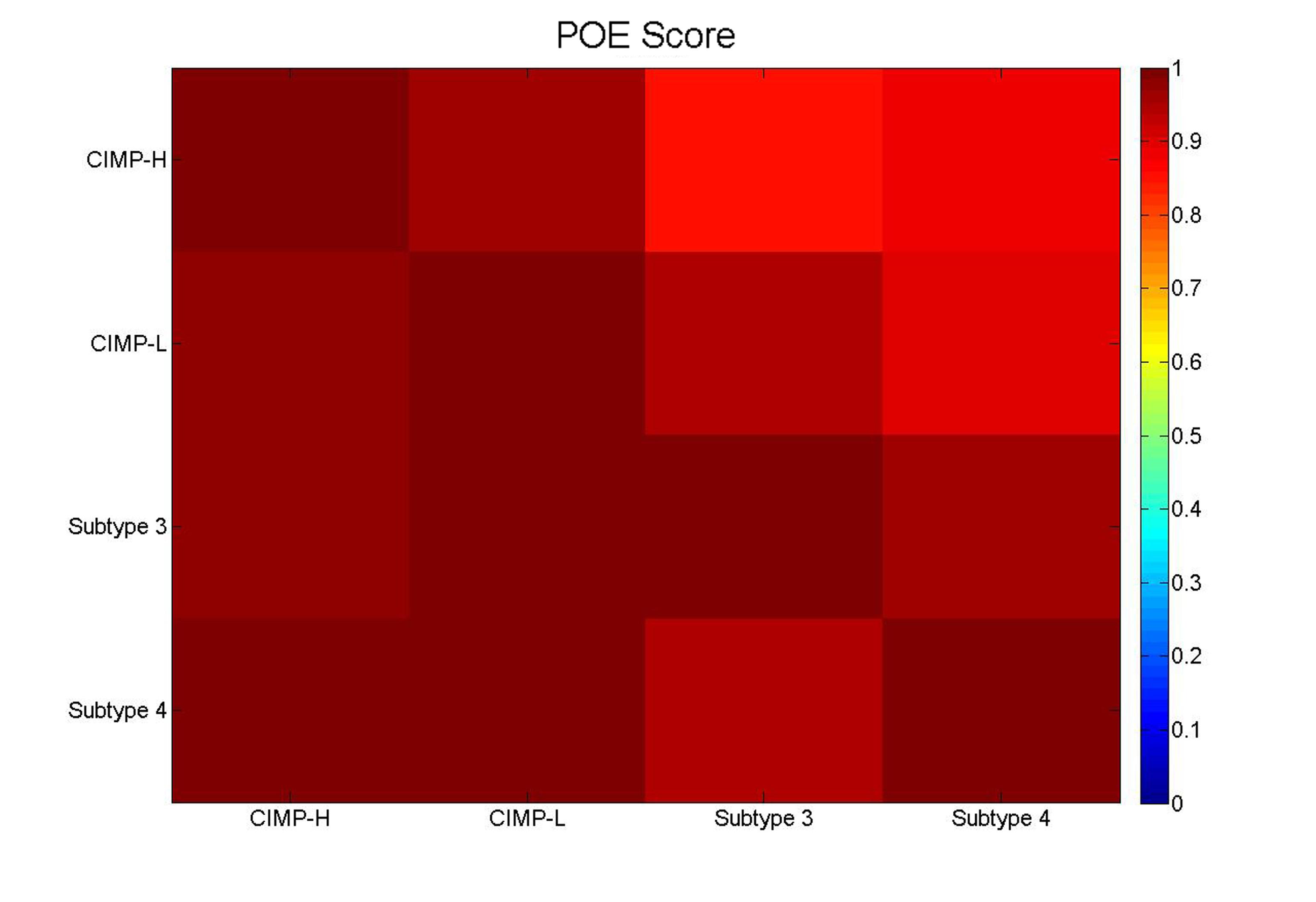

Supplement: Figure S1 — The POE scores between every two lists of the hypermethylated functions extracted for the four methylation-based subtypes of colon cancer. Each row represents the scores between the hypermethylated terms for one subtype and the hypermethylated terms for the other subtypes. The POE score 1 is shown in red and 0 is indicated in blue. The details of the four subtypes are described in [35]. (TIF) [file pone.0044822.s001.tif]
